# The Slovenian version of the Cardiac depression scale – validity and reliability

**DOI:** 10.1192/j.eurpsy.2024.546

**Published:** 2024-08-27

**Authors:** A. Kokalj Palandacic, S. Ucman, M. Lainscak, B. Novak Sarotar

**Affiliations:** ^1^University Psychiatric Clinic Ljubljana; ^2^Faculty of Medicine, University of Ljubljana, Ljubljana; ^3^Department of Internal medicine, Cardiology devision, General Hospital Murska Sobota, Murska Sobota, Slovenia

## Abstract

**Introduction:**

Cardiovascular diseases (CVD) were the cause of 40% of all deaths in Slovenia in 2016, and are the seventh most common cause of visits to the general practitioner. The prevalence of depression in people with CVD is high and is a strong predictor of mortality and additional cardiac events. In patients with coronary artery disease, depressive symptoms contribute to a lower quality of life and to physical limitations.

**Objectives:**

The purpose of this study was to translate the Cardiac Depression Scale into Slovenian (S-CDS) and to assess its psychometric properties on Slovenian patients with heart disease.

**Methods:**

After obtaining the consent from the original authors, the Cardiac depression scale was translated by three bilingual Slovenian native speakers with medical knowledge. Afterwards, they worked jointly to reach consensus on one version, which was then back-translated (Slovenian to English) by two independent English translators unfamiliar with the original version. The original authors approved the final draft. The S-CDS was then applied to a total of 272 patients with heart disease that underwent elective coronary angiography. At the same time the Spielberger Stait Anxiety Inventory (STAI-S) and the Center for Epidemiologic Studies Depression Scale-20 (CES-D) were used. An exploratory and confirmatory factor analysis, internal consistency, test–retest reliability and concurrent validity were performed.

**Results:**

The total scale had Cronbach’s alpha 0.92 and test–retest reliability 0.71. Six factors were confirmed by the exploratory factor analysis, accounting for 60.88% of total variance. A two and one factor solution indicated by the confirmatory factor analysis had acceptable goodness-of-fit measures. A one factor solution was kept, considering a high correlation between the two factors and the theoretical background in previous studies. A moderate to strong correlations were confirmed by concurrent validation against the CES-D and the STAI-S.

**Conclusions:**

The S-CDS with 25 questions is a reliable and valid instrument for measuring depressive symptoms in Slovenian patients with heart disease.

**Disclosure of Interest:**

None Declared

